# Practical applications of copper-based enzymes: synthesis of sulfonated polyaniline through laccase-catalyzed oxidation

**DOI:** 10.3389/fchem.2024.1412242

**Published:** 2024-07-25

**Authors:** Fabrizia Fabrizi de Biani, Maddalena Corsini, Rebecca Pogni, Maria Camilla Baratto

**Affiliations:** ^1^ Dipartimento di Biotecnologie, Chimica e Farmacia, Università di Siena Via Aldo Moro, Siena, Italy; ^2^ INSTM, Research Unit of Siena, Siena, Italy; ^3^ Centre for Colloid and Surface Science (CSGI), Firenze, Italy

**Keywords:** laccase, sulfonated polyaniline, 3-aminobenzenesulfonic acid, cyclic voltammetry, spectroelectrochemistry, conducting polymers

## Abstract

Known for its tunable conductivity and stability, Polyaniline (PANI) is a valuable polymer for electronics and sensing devices. Challenges in solubility have been addressed by creating sulfonated PANI (SPANI), enhancing its practical use. Synthesizing SPANI from sulfonated aniline is intricate, but laccase biocatalysis offers an eco-conscious solution, effective even against high redox potential obstacles. This research monitored the *Trametes versicolor* laccase-induced oxidation of 3-ABSa via UV-vis spectroscopy, with a notable peak at 565 nm signifying SPANI synthesis, effective even at suboptimal pH. Mediators further boost this process. Moreover, NMR and spectroelectrochemistry confirm the green synthesis of SPANI by laccase, hinting that pH fine-tuning could improve yields, alongside the concurrent creation of azobenzene derivatives.

## 1 Introduction

**FIGURE 1:**
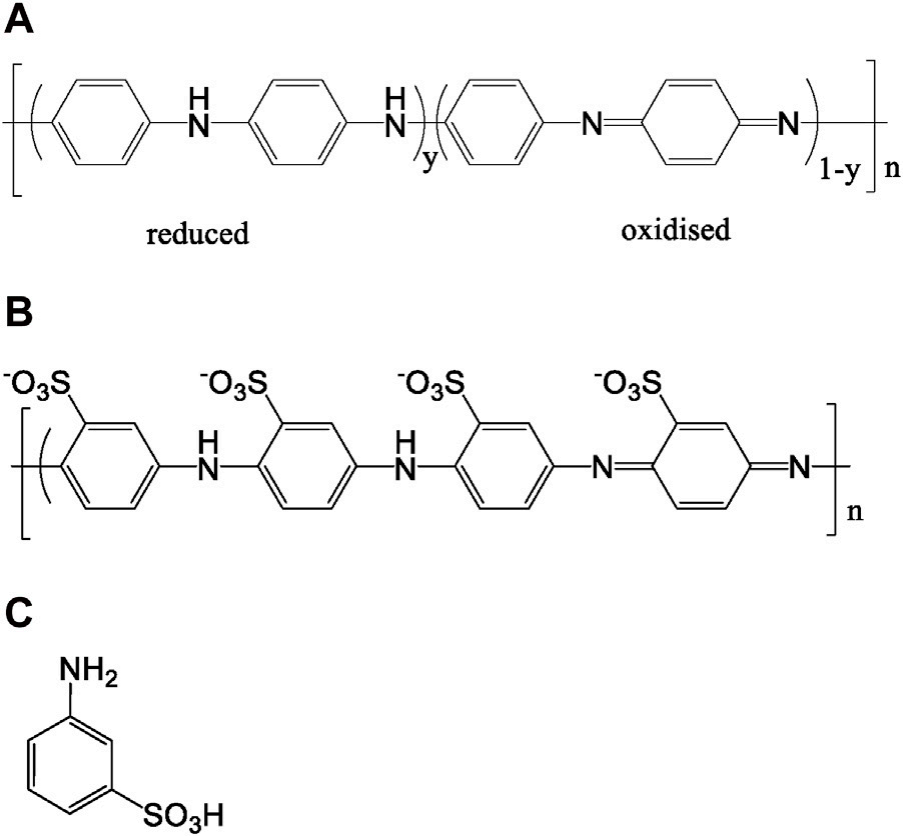
**(A)** Schematic polyaniline (PANI). The fully reduced Leucoemeraldine (x = 0, y = 1), half oxidised Emeraldine (x = y = 0.5) and fully oxidised Pernigraniline (x = 1, y = 0) forms; **(B)** Sulfonated polyaniline (SPANI); **(C)** 3-aminobenzenesulfonic acid (3-ABS).

Polyaniline (PANI) is a conducting polymer that has been of interest since the 1980s due to its unique properties. It is one of the most studied conducting polymers due to its ease of synthesis, low cost, stability, and wide range of conductivity upon doping (Bhadra et al., 2020; Majeed et al., 2022). Polyaniline and similar oligomers/polymers serve as highly functional materials across various domains. Recent instances highlight the potential application of PANI composite materials in designing wearable electronics (Lyu et al., 2020; Zheng et al., 2023; Chen et al., 2023), for environment remediation (Lyu et al., 2021; Lyu et al., 2022) and for seawater desalination (Han et al., 2022), furthermore PANI-supported nano metal particles have been broadly applied as catalyst in coupling reactions (Zeng et al., 2023). The base form of PANI has a generalized composition as in Figure 1A. Leucoemeraldine is the fully reduced state of the polymer, it exists in the colorless base form. Emeraldine is the half-oxidized state of the polymer, it can be found in both the base form (blue) and the protonated “salt” form (green). Pernigraniline is the fully oxidized form of the polymer, it can also exist in both the base form (violet) and the salt forms (blue). One of the key properties of PANI is its adjustable electrical conductivity: the conductivity of PANI depends on its oxidation state, furthermore, it can be increased by up to ten orders of magnitude when treated with acids (Majeed et al., 2022). Since PANI also exhibits color changes associated with different oxidation states, it can be also used in sensors and electrochromic devices (Majeed et al., 2022). Furthermore, PANI has excellent environmental stability, making it suitable for use in various conditions. In summary, PANI is a versatile conducting polymer with unique properties that make it suitable for a wide range of applications, however, limitations such as its poor solubility in common solvents and its high melting point have impeded its incorporation into industrial applications. To overcome these limitations, design strategies to enhance the solubility of PANI in an aqueous solution without adversely impacting conductivity and electroactivity have been developed over the years and various approaches have been pursued to enhance the solubility by the introduction of substituents on the PANI backbone. In 1990, Jiang et al. and Dao et al. reported the first water-soluble conducting derivatives of PANI, i.e. sulfonated polyaniline (SPANI) (Jiang and Epstein, 1990; Bergeron et al., 1990, Figure 1B). The process of sulfonating PANI using fuming sulfuric acid involves dealing with hazardous and corrosive chemicals, which makes it suboptimal and challenging to expand on a larger scale. Furthermore, the gradual dissolution of PANI in concentrated sulfuric acid is manifest. This is likely due to a simultaneous reaction with SO_3_, resulting in complicated reaction patterns including partial sulfonation of the polymer and multiple sulfonation on the same ring (Yue et al., 1992). Despite the difficulties, sulfonated polyanilines initially have been typically synthesized through post-polymerization treatment of PANI. Several research groups have attempted to homopolymerize sulfonated aniline, but this has proven to be a non-trivial task and initial efforts to directly polymerize ring-sulfonated aniline were unsuccessful. This outcome has been attributed to the combination of the steric inhibition and the electron-withdrawing nature of the sulfonic acid group, which reduces the monomer’s susceptibility to oxidation (Yue et al., 1991). The first chemical homopolymerization of 3-aminobenzenesulfonic acid (3-ABSa, Figure 1C) was accomplished (in low yield) by conducting the reaction under high pressure (Chan et al., 1998). Subsequently, polymerization of 3-ABSa was carried out under ambient pressure using FeCl_3_·6(H_2_O) as both a binary oxidant and dopant agent. This reaction occurred in a solvent-free condition at temperatures ranging from 40°C to 45°C. Under these conditions, the fully oxidized pernigraniline form was successfully obtained (Modarresi-Alam et al., 2019). In their study, Mav et al. found that 3-ABSa does not homopolymerize through chemical oxidative polymerization in water at low pH (Mav et al., 1999). Also, when they prepared a series of copolymers using aniline and 3-ABSa with varying ratios of comonomers, they noticed a decrease in yield as the proportion of the less reactive sulfonated monomer increased in the reaction mixture. This observation aligned with the previously reported expectation that sulfonated aromatic rings should be isolated by at least one non-sulfonated aromatic ring in the structure of PANI (Yue et al., 1991). Despite these obstacles, electrochemical polymerization of 3-ABSa has eventually been performed either by the controlled current (Kitani et al., 1995) or by controlled potential (Márquez et al., 2007) conditions or by cycling voltammetry in a solvent mixture CH_3_CN/H_2_O (Krishnamoorthy et al., 2002). In summary, even if there have been some fruitful attempts, the homopolymerization of ortho-, meta- and para-aminobenzenesulfonic acid (2,3,4-ABS) remains a challenging endeavor (Freund and Bhavana, 2007). On the other side, biocatalysis has emerged as an environmentally friendly alternative for synthesizing PANI (Karamyshev et al., 2003; Walde et al., 2019; De Salas et al., 2016). Biocatalysis offers advantages such as milder processing conditions (neutral pH and lower temperature) and reduced waste and toxicity, aligning with the principles of green chemistry (Clark, 1999). Laccase, a multicopper oxidoreductase, can catalyze the oxidation of many substrates, with atmospheric oxygen as the oxidizing agent. In the following, we illustrate the uncomplicated process for obtaining SPANI through the polymerization of the monomeric unit 3-ABSa facilitated by the catalytic activity of laccase. The redox potential of the laccases is in the range of +0.40 to +0.80 mV (vs NHE) and specifically *Trametes versicolor* laccase has a redox potential of +0.78 mV (Reinhammar, 1972; Xu et al., 1996). The pH-dependent redox potential of 3-ABSa is expected to be remarkably elevated, posing a challenge for laccase in its oxidation process. It has been previously reported (Polak and Jarosz-Wilkołazka, 2010) that at pH 4.5 the redox potential of 3-ABSa is +1.18 V (vs NHE) and therefore it should be not oxidized by *Trametes versicolor* laccase. Anyway, there are evidence that laccases can indeed oxidize compounds with a higher redox potential than their own, although the precise mechanism behind this phenomenon remains unclear (Amann, 1997; Bourbonnais et al., 1998). In our experimental observations, we encountered a similar outcome: *Trametes versicolor* laccase catalyzed the formation of a red-purple-colored polymer from 3-ABSa within a pH 4.6 buffered solution. Although not entirely surprising, this fascinating result is entangled by the inherent nature of the substrate, which opens the way to an array of diverse potential oxidation products.

## 2 Materials and methods

3-Aminobenzensulphonic acid (3-ABS, 97%), laccase from *T. versicolor* (≥0.5 U/mg), 2,2’-azino-bis(3-ethylbenzothiazoline-6-sulphonic acid (ABTS, 98%), methylsyringate (98%) and acetylacetone (98%) were purchased by Sigma-Aldrich. All solutions were prepared in deionized H_2_O and in acetate buffer 0.1 M at pH = 4.6. The UV-Vis spectrophotometric measurements were conducted using a Perkin Elmer Lambda 900 instrument within the wavelength range of 200–900 nm. A Helma Certified quartz suprasil 10 mm cuvette was employed for the measurements. The reaction was monitored every 15 min for 3 h and then after 24 h. To achieve high-quality spectra, the solutions needed to be diluted fivefold due to the high extinction coefficient of the emerging absorption. Lyophilization and Chromatographic Separation: to determine the yield of the dye formation after 3 h and after 24 h and to perform the IR study of the dried product, the dye solution was lyophilized using a Lio5P freeze dryer. The solutions were lyophilized for 24 h. IR spectra of the lyophilized samples were recorded using an Agilent Cary 630 FTIR Instrument. 1H NMR spectra were recorded at 400 MHz with a Bruker Advance DPX400. Cyclic voltammetry was conducted using a BAS 100 W potentiostat. The experiment employed a low-volume electrochemical cell equipped with an Ag/AgCl (3 M) Reference electrode, a Pt wire Counter electrode and a glassy carbon Working electrode. All the potential values have been converted to use NHE as a reference potential. Spectroelectrochemistry was performed using a quartz cuvette with an optical path length of 1 mm and using a Pt mesh as working electrode.

## 3 Results and discussion

### 3.1 UV-vis spectroscopy

**FIGURE 2:**
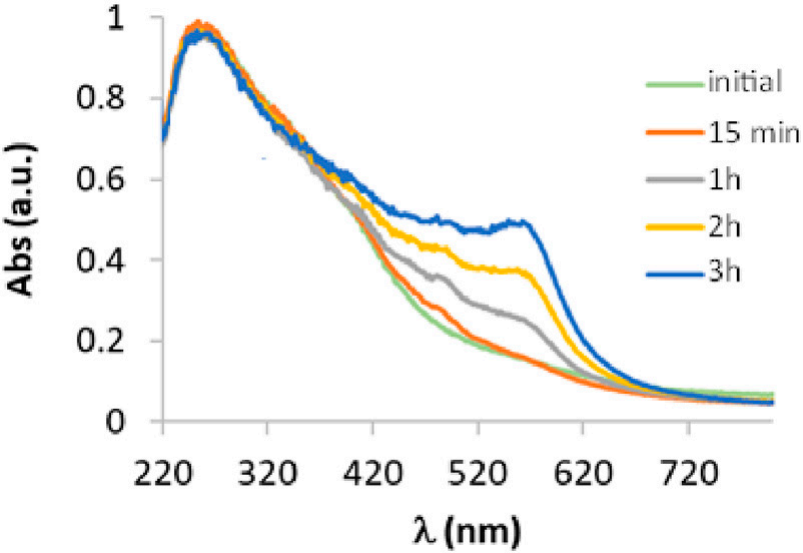
UV-vis spectra of a solution containing 3-ABSa (13.2 mM) and laccase from *Trametes versicolor* (66 μM) at pH 4.6 (acetate buffer 0.1 M) monitored at 1 min, 15 min, 1 h, 2 h, and 3 h after the enzyme addition.

**FIGURE 3:**
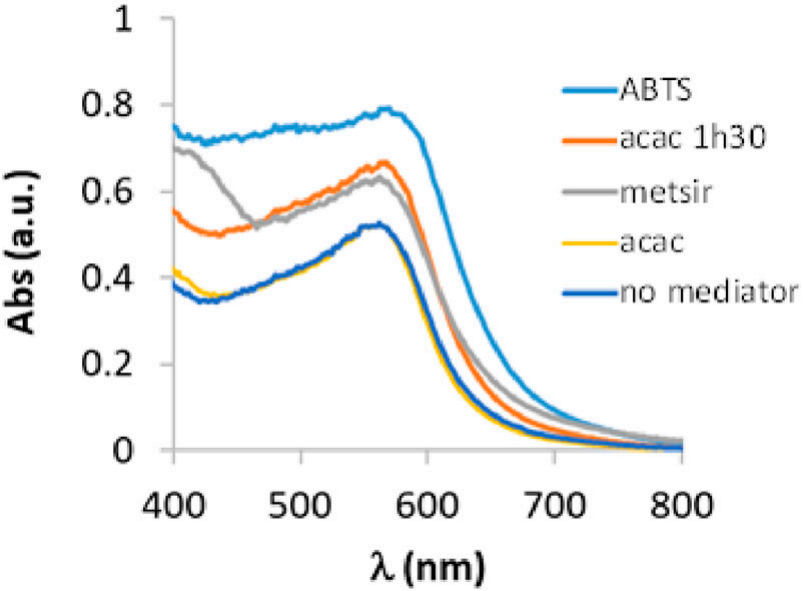
UV-vis spectra of a solution containing 3-ABSa (13.2 mM), laccase from *Trametes versicolor* (66 μM) at pH 4.6 (acetate buffer 0.1 M) (black line) and ABTS (red line), acetylacetone (acac, blue line), acetyleacetone in keto-enolic equilibrium (acac 1 h 30, yellow line) and methylsiringate (metsir, green line) monitored after 24 h of reaction.

To evaluate the catalytic activity of laccase in the oxidation of 3-ABSa, we have mixed a solution of 3-ABSa (13.2 mM) in a 0.1 M acetate buffer at pH 4.6 with a solution of laccase (66 μM) at the same pH. The molar ratio of laccase to precursor was therefore 1:200. Throughout the experiment, we continuously monitored the reaction using UV-vis spectroscopy at regular 15-minute intervals over 3 h, followed by a final assessment after 24 h. The initial spectra (up to 3 h of reaction) are depicted in Figure 2. Clearly, the most prominent feature is the emergence of a band at 565 nm, which steadily intensifies. Additionally, there is a visible band at 300 nm with a broad shoulder at ∼380 nm, and its intensity remains relatively constant. Furthermore, there is a shoulder around 410 nm that gradually increases. Since it is well-established that the catalytic activity of laccase can be expanded to a broader range of substrates by employing mediators, we also investigated the influence of the presence of three possible mediators: ABTS, acetylacetone, and methylsyringate, on the oxidation process. Moreover, in the case of acetylacetone we repeated the experiment using a solution that had been previously allowed to rest for 1 h and 30 min to reach the keto-enolic equilibrium. This allowed us to observe the impact of the keto-enolic equilibrium on the mediator efficiency. In all cases, the mediators were present at a concentration of 0.66 mM, resulting in a laccase:mediator:substrate ratio of 1:10:200. In Figure 3, we compare the UV-vis spectra of reactions in the presence of these mediators. Specifically, we examine the spectra obtained in the presence of the laccase-mediator (mediators: ABTS, acetylacetone, methylsyringate) with the reaction run solely in the presence of the precursor without any mediator. The high energy region of the spectra shows slight differences for each of the experiments, primarily because this is the region where the mediators have their own absorptions. However, after 24 h, all the spectra consistently display a prominent band at 565 nm, suggesting that the product formed remains consistent across all cases. Except when non-equilibrated acetylacetone was used, the presence of mediators results in a higher intensity of this band, indicating a certain level of efficiency for the mediators. Although the impact of mediators on the reaction yield is apparent, it is important to recognize that the reaction still occurs even in their absence. Consequently, to streamline the product characterization process and prevent interference from mediators, we exclusively investigated the laccase-precursor reaction for the remainder of our study.

### 3.2 FTIR spectroscopy

**FIGURE 4:**
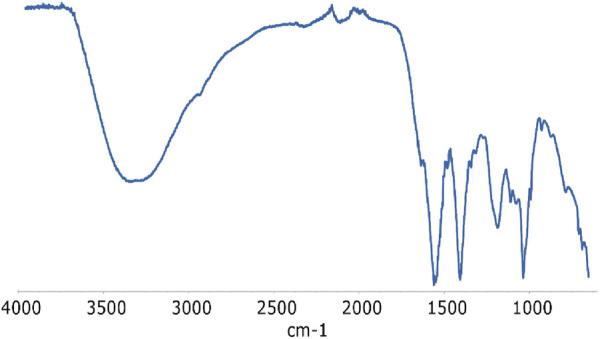
IR spectrum of the lyophilised solution after 24 h of reaction.

Regrettably, infrared spectroscopy did not prove very useful in identifying all the relevant functional groups within the lyophilized product for characterization. The IR spectrum (Figure 4) is largely influenced by signals arising from sodium acetate, which originates from the acetate buffer. Therefore, the stretching vibrations associated with the carboxylic group of sodium acetate (at 1,554 and 1,403 cm^-1^) dominate a critical region of interest for characterizing PANI compounds and somehow obscure the bands of quinonoid ν(C = C)_Q_ and benzenoid ν(C = C)_B_ ring stretching vibrations expected at ∼1,560 and 1,490 cm^-1^, respectively. The aromatic region (typically characterized by intense and sharp signals) in the 600–900 cm^-1^ range also appears ambiguous. Anyway, the presence of the SO_3_^−^ group is revealed by the peaks at 1,185 and 1,034 cm^-1^, which may be assigned to symmetric and asymmetric stretching of the S=O group (Krishnamoorthy et al., 2002). ν(N–H) stretching modes are also visible around 3,350 cm^-1^ and give a relevant indication on the oxidation state of the compound, since these modes are consistent with the partially oxidized (emeraldine) form of the polymer, while they are not present in the fully oxidized (pernigriline) form.

### 3.3 ^1^H-NMR measurements

**FIGURE 5:**
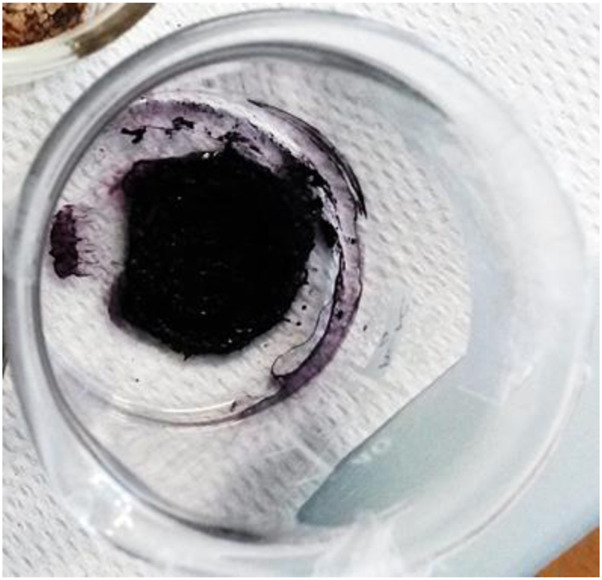
Appearance of the lyophilised solution after 24 h of reaction.

**FIGURE 6:**
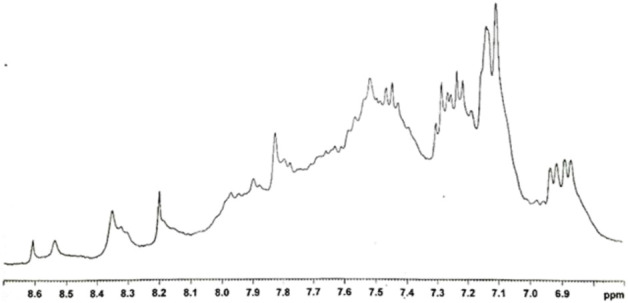
^1^H NMR spectra of the lyophilised solution after 24 h of reaction.

**FIGURE 7:**
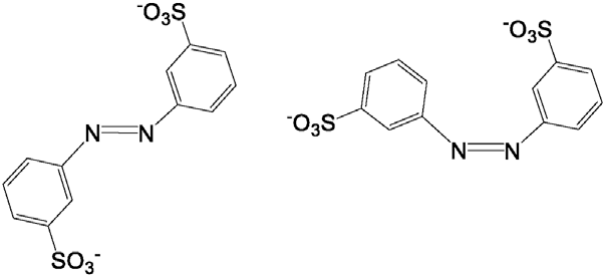
3,3-disulfonate azobenzene in its *cis* and *trans* form.

**FIGURE 8:**
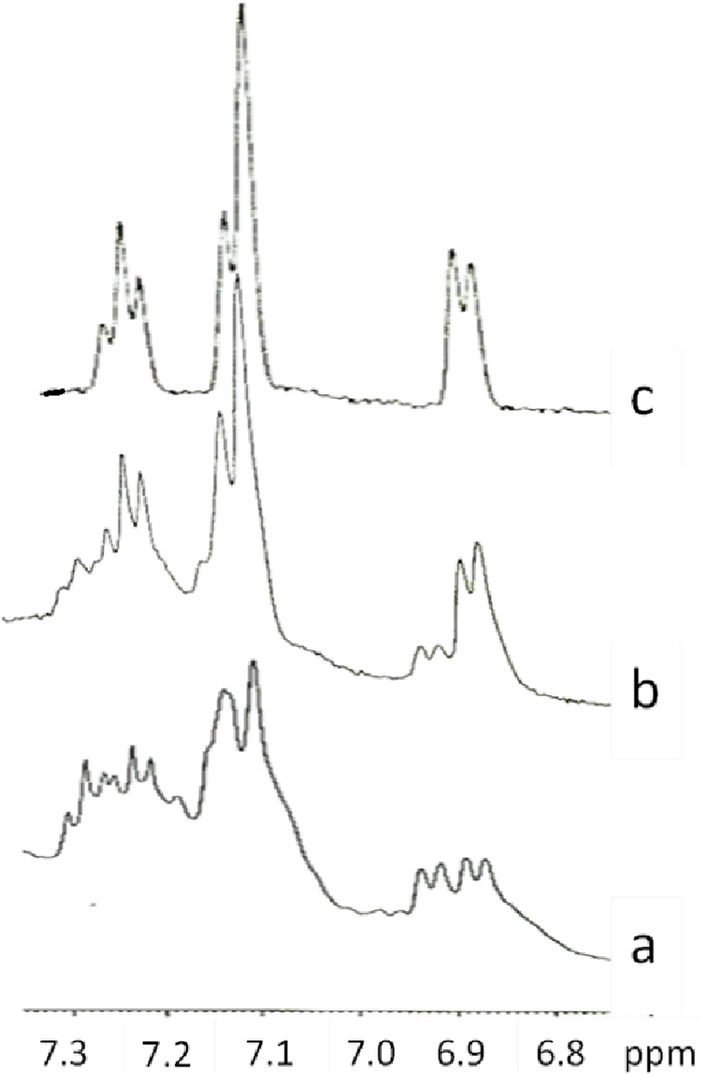
The ^1^H NMR spectra of the lyophilized solution after 24 h of reaction in the original state **(A)**, after Irradiation at 380 nm **(B)**, after heating at 120°C **(C)**.

To get dry samples appropriate for the preparation of solutions in deuterated water, the purple solution has been lyophilized (Figure 5) and subsequently heated. The heating temperature was decided after having checked the behavior of the compound upon heating. Thus, samples obtained after different reaction times were tested to define a melting point. We observed that all the samples, however obtained, darken at around 200°C and it is not possible to detect a precise melting point. Given these considerations, to prevent samples decomposition, we opted to desiccate them by heating at 120°C (for 15 h). However, heating to this temperature also produced a change in the color of the samples. It is noteworthy that when the reaction between 3-ABSa and laccase was interrupted after 3 h, heating induced a significant color alteration in the resulting sample, which shifted from purple to brown-yellow. However, when the reaction had continued for 24 h, the color variation upon heating was less pronounced, transitioning from purple to brownish-violet. Therefore, we have further investigated the NMR analysis of the most stable product, obtained after 24 h of reaction. In Figure 6, we present the ^1^H NMR spectrum of the purple sample obtained after 24 h of reaction, lyophilized, but not heated. The presence of two evident doublets at 6.87–6.91 ppm is highly suggestive of the presence of two isomeric forms. In a more precise interpretation, the region from 6.8 to 7.4 ppm can be interpreted as due to overlapping patterns arising from the signals of two isomers, both featuring a meta-aromatic substitution. Each of these overlapping patterns consists of the following components: a doublet, a singlet overlapping a doublet and a triplet. These features strongly suggest the presence of 3,3’-bis(sulfonate) azobenzene in its *cis* and *trans* forms (Figure 7). The remaining portion of the NMR spectrum consists of irregular peaks that are challenging to characterize, as is typical of polymers. To further investigate the presence of azobenzene isomers, which often exhibit photoisomerization capabilities, we irradiated this sample with two different wavelengths, selected from those corresponding to the maximum absorption in the UV spectrum. We observed that while irradiation with a wavelength of 550 nm has no effect, irradiation at 380 nm leads to the gradual increase of the doublet signal at 6.87 ppm, at the expense of the signal at 6.91 ppm, which decreases (Figure 8). Furthermore, the spectral region between 7.0 ppm and 7.4 ppm reveals a significantly simplified structure, with the distinctive pattern of a meta-substituted aromatic compound (two doublets, one singlet and one triplet) becoming distinctly apparent. When heating the sample at 120°C, we observe a similar effect, as depicted in Figure 8C and the ^1^H NMR spectrum confirms the existence of only one isomer. It is important to note that only the discussed region of the ^1^H NMR spectrum exhibits alterations, while the remaining part of the spectrum remains unchanged. This behavior suggests that in the sample obtained through the oxidation of 3-ABSa catalyzed by laccase over 24 h, there is the simultaneous presence of two compounds, one of which is photoisomerizable. It is reasonable to assume that these compounds are 3,3’-bis(sulfonate) azobenzene (in both *cis* and *trans* forms) and a polymer of sulfonated aniline, SPANI.

### 3.4 Electrochemistry and spectroelectrochemistry

**FIGURE 9:**
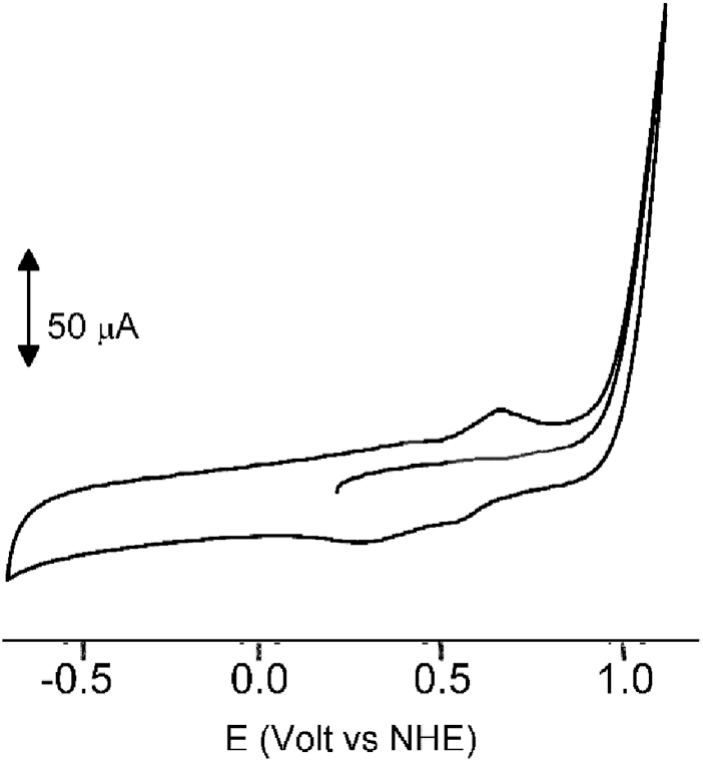
The cyclic voltammogram of a solution of 3ABSa (5.2 × 10–^3^ M) in water at pH 4.6 (acetate buffer 0.1 M used as electrolyte), Scan rate 0.2 V s^-1^.

**FIGURE 10:**
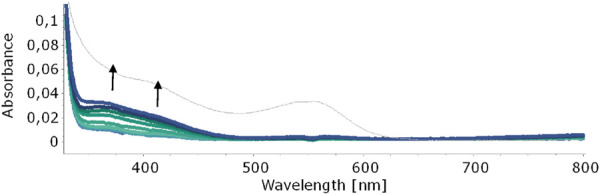
UV-vis spectra collected upon oxidation of a solution of 3-ABSa (pH 4.6, acetate buffer 0.1 M). Initial potential +0.4 V, final potential +0.7 V. The thin gray line shows the sudden change of the spectrum as soon as the potential +0.7 V is overcome.

**FIGURE 11:**
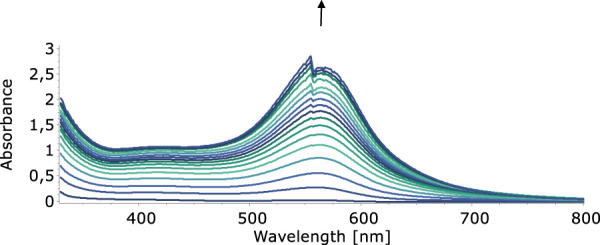
UV-vis spectra collected upon oxidation of a solution of 3-ABSa (pH 4.6, acetate buffer 0.1 M). Initial potential +0.75 V, final potential +1.45 V.

**FIGURE 12:**
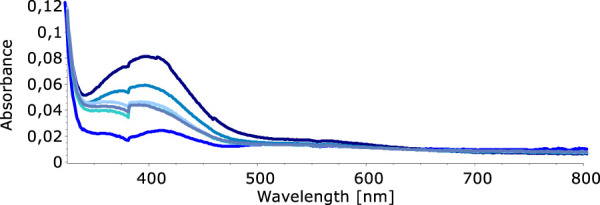
UV-vis spectra collected upon oxidation of a solution of 3-ABSa (pH 7, NaCl 0.1 M as the electrolyte). Initial potential + 0.4 V, final potential +1.45 V.

In our quest to understand the oxidation mechanism of 3-ABSa and to characterize the resulting species, we conducted electrochemical and spectroelectrochemical measurements. During these experiments, we induced the oxidation of 3-ABSa without laccase, keeping all other experimental conditions constant. Figure 9 shows the cyclic voltammogram of an aqueous solution of 3-ABSa (pH = 4.6, acetate buffer as electrolyte). During the initial anodic potential scan, we observed an irreversible oxidation process near the point of solvent discharge. This phenomenon appears as an offset at + 0.9 V. Interestingly, immediately after surpassing this potential and initiating the oxidation of 3-ABSa, a purple coloration became visible near the electrode. As the positive potential scan continued, the species diffused into the bulk solution, eventually causing the entire solution to turn purple. On the back scan, around + 0.55 V, we observed a cathodic peak, likely corresponding to the reduction of the recently formed and transient radical cation 3-ABSa^⋅+^. On the other hand, the cathodic current peak observed at + 0.35 V is attributed to the reduction of the oligomeric or polymeric chains of sulfonated polyaniline, which then undergo an oxidation at + 0.65 V. During potential cycling, the redox peaks associated to the redox change of SPANI oligomers/polymers exhibited only a slight increase and we did not observe the electrodeposition of a film on the electrode surface. Instead, colored soluble oligomeric/polymeric products were observed streaming away from the electrode surface. In Figures 10, 11, we show the UV-vis spectra obtained during the spectroelectrochemical analysis of 3-ABSa at pH 4.6. This analysis involved collecting spectra while varying the potential from + 0.4 V to + 1.45 V. As the potential approaches +0.7 V, we clearly observe two overlapping bands at 370 nm and 410 nm, which exhibit slight growth (see Figure 10). However, when the potential reaches + 0.9 V, a broad and extremely intense band centered at 565 nm suddenly appears and dominates the spectral aspect (see Figure 11). When the experiment is repeated at pH = 7 with NaCl as the electrolyte, we observe the following spectral behavior upon oxidation: the bands at 370 nm and 410 nm appear, while the band expected at 565 nm is dramatically diminished and is almost never visible (Figure 12). This intriguing behavior can be elucidated by considering that the species formed during the oxidation of 3-ABSa at pH = 7 may also be the same species that forms in the initial phase of oxidation at pH = 4.6. This species likely corresponds to sulfonated azobenzene. On the other hand, during more vigorous oxidation at pH = 4.6, the resulting species likely consists of purple-colored SPANI oligomers.

## 4 Discussion

**FIGURE 13:**
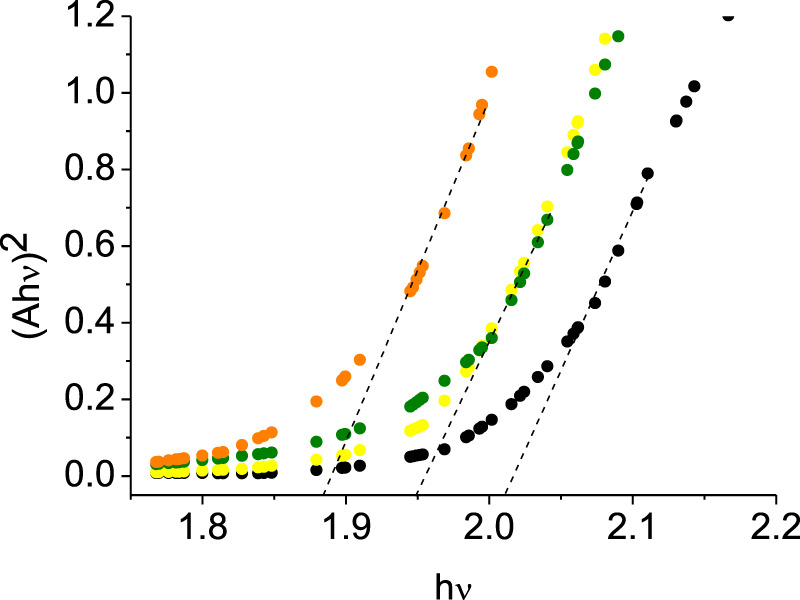
Tauc plot obtained for the band with λ = 565 nm. Compound obtained in absence of a mediator (black dots), in the presence of metsir (green dots), acac1h30 (yellow dots), ABTS (orange dots).

**FIGURE 14:**
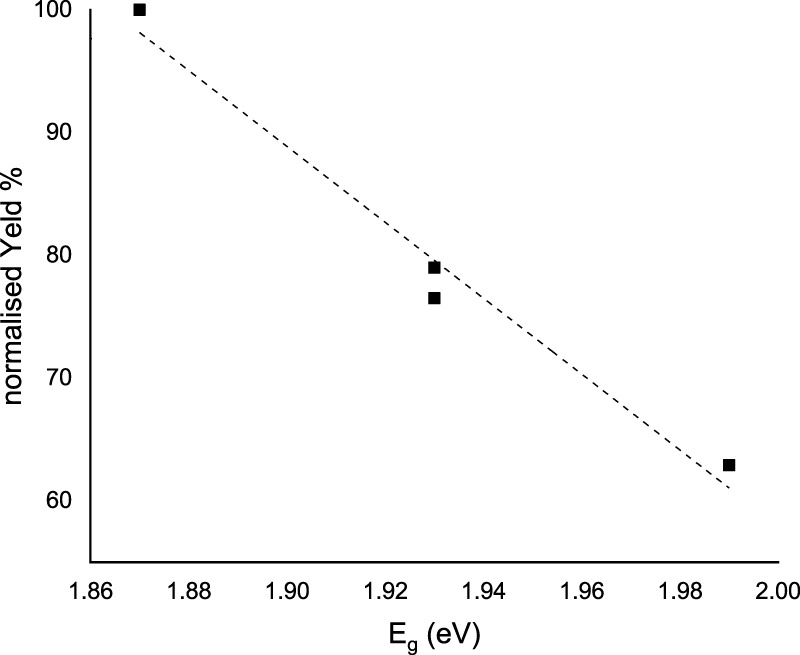
Linear correlation between E.g., (eV) of the polymeric SPANI obtained in the presence of different mediators and the normalized yield (%).

**FIGURE 15:**
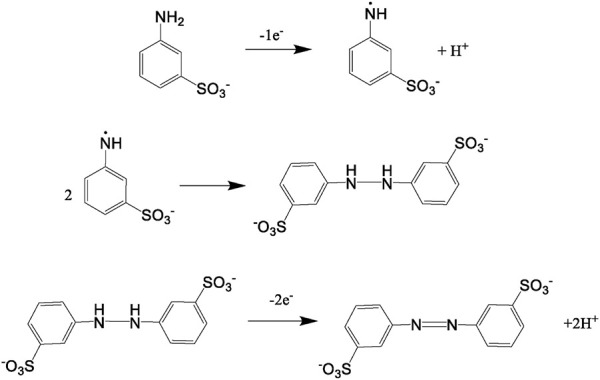
The formation of 3,3’-bis(sulfonate) azobenzene releases 3H^+^.

When 3-ABSa undergoes oxidation, whether through enzymatic processes or electrochemistry, it produces a purple product characterized by a broad peak around 565 nm. Previous studies have associated this absorption band with an exciton-like transition occurring in the quinoid diimino units of the conducting semioxidized (emeraldine) form (Krishnamoorthy et al., 2002; Madhurima et al., 2019). Additionally, there is another absorption band observed at ∼300 nm, which is attributed to a π–π* transition in benzenoid units. Remarkably, during spectroelectrochemical experiments, we observe spectra that reveal additional bands at 370 nm and 410 nm. These bands are typically not present in previous studies on SPANI, especially those conducted in strongly acidic media. Later, we will delve into more detail, but it is reasonable to associate these two bands with the presence of 3,3’-bis(sulfonated) azobenzene. Focusing specifically on the absorption band at 565 nm, it is noteworthy that in the presence of mediators, this band experiences a slight blue shift following the order: λmax (no-mediator) ≈ acac < metsyn ≤ acac1h30 < ABTS. This shift could be attributed to the improved orientation and an associated increase in the conjugation length of the SPANI chain. To determine the optical absorption band gap (E_.g.,_) of the polymer obtained under different experimental conditions, we employed the Tauc relation, assuming a direct and allowed electronic transition (Tauc, 1972):

$${\left(Ah\nu \right)}^{2}=k\left(h\nu \;-\;E_{g}\right)$$

where A is the Absorption (in arbitrary units) and k is a constant. Figure 13 shows the plot of (Ahν)^2^ vs. hν. The estimated band gap values range from 2.00 eV (SPANI obtained without a mediator or with acac) to 1.87 eV (SPANI obtained in the presence of ABTS). The reduction in the band gap implies that the presence of a mediator promotes the aligned arrangement of SPANI chains. This phenomenon appears to be linked to the efficiency of the SPANI formation process. As depicted in Figure 14, the optical band gap values (E_.g.,_) exhibit a positive correlation with the SPANI yield obtained under various conditions (normalized with respect to the maximum yield, achieved in the reaction with ABTS). Although the precise reason behind this correlation remains undisclosed, it is highly plausible that a well-organized and highly conjugated polymer would yield superior outcomes. Having confirmed the presence of SPANI, spectroelectrochemistry has proven to be particularly useful in revealing the formation of 3,3’-bis(sulfonated) azobenzene as a byproduct. Notably, the spectra collected during oxidation at pH 4.6 exhibit an initial evolution that differs from what is observed as the potential becomes more positive. According to the literature (Desideri et al., 1973), when the pH is only moderately acidic, the oxidation of aminobenzenesulfonic acids predominantly leads to the formation of sulfonated azobenzene compounds. This occurs via a radical intermediate, as depicted in Figure 15. Therefore, a plausible interpretation of the behavior observed through spectroelectrochemistry is that at the outset, the reaction begins under mildly acidic conditions and 3,3'-bis(sulfonated) azobenzene (bands at 370 and 410 nm) initially forms as a byproduct of the oxidation of 3-ABSa. However, as the process unfolds, the release of 3 H^+^ ions from the oxidation of 3-ABSa lower the local pH shifts into the high acidity region. and 3-ABSa oligomers (band at 565 nm) emerge in the advanced stage, when the local acidity reaches a critical level. This hypothesis is confirmed by the spectroelectrochemical experiment conducted at pH = 7, which reveals the appearance of only the bands around 380 nm and 410 nm. This gives indication that at this specific pH value, only the azobenzene derivative forms. Finally, the presence of 3,3’-bis(sulfonated) azobenzene in its two isomers, which forms alongside SPANI, is confirmed by NMR experiments and irradiation experiments. These experiments demonstrate a selective variation in one part of the spectrum due to irradiation, while another part remains unchanged. This behavior aligns with what one would expect from a mixture of SPANI and azobenzene derivatives.

## 5 Conclusion

In conclusion, we have demonstrated that sulfonated polyaniline polymer SPANI can be easily obtained through the oxidation of 3-ABSa, catalyzed by laccase from *T. versicolor*. This process can be carried out under environmentally friendly conditions, using water at moderate pH values, room temperature and without the need for additional reagents. However, SPANI is not obtained as a sole product; rather, its formation is accompanied by 3,3’-bis(sulfonated) azobenzene. It is reasonable to think that achieving a higher production of SPANI could be possible by operating at a lower pH. However, excessively low pH values are incompatible with laccase enzymes: even fungal laccases, which typically perform better in acidic conditions, have their optimal pH value around 4 (Arregui et al., 2019). In the future, investigating optimal pH values to maximize SPANI production and exploring how these pH values impact the length of polymer chains could yield valuable insights.

## Data Availability

The original contributions presented in the study are included in the article/Supplementary Material, further inquiries can be directed to the corresponding author.
